# Epigenetic regulation of B cells and its role in autoimmune pathogenesis

**DOI:** 10.1038/s41423-022-00933-7

**Published:** 2022-10-12

**Authors:** Fan Xiao, Ke Rui, Xiaofei Shi, Haijing Wu, Xiaoyan Cai, Kathy O. Lui, Qianjin Lu, Esteban Ballestar, Jie Tian, Hejian Zou, Liwei Lu

**Affiliations:** 1 Department of Pathology, Shenzhen Institute of Research and Innovation and Shenzhen Hospital, The University of Hong Kong, Hong Kong; Chongqing International Institute for Immunology, Chongqing, China; 2 Institute of Medical Immunology, Affiliated Hospital of Jiangsu University, Zhenjiang, China; 3 Department of Rheumatology and Immunology, The First Affiliated Hospital and School of Medicine, Henan University of Science and Technology, Luoyang, China; 4 Department of Dermatology, Second Xiangya Hospital, Central South University, Hunan Key Laboratory of Medical Epigenomics, Changsha, Hunan China; 5 Department of Rheumatology, Guangzhou First People’s Hospital, School of Medicine, South China University of Technology, Guangzhou, China; 6 Department of Chemical Pathology, Faculty of Medicine, Prince of Wales Hospital, The Chinese University of Hong Kong, Hong Kong, China; 7 Epigenetics and Immune Disease Group, Josep Carreras Research Institute, Badalona, 08916 Barcelona, Spain; 8 Epigenetics in Inflammatory and Metabolic Diseases Laboratory, Health Science Center, East China Normal University, Shanghai, China; 9 Department of Rheumatology, Huashan Hospital, Fudan University, Shanghai, China; 10 Centre for Oncology and Immunology, Hong Kong Science Park, Hong Kong, China

**Keywords:** B cells, Epigenetic regulation, Autoimmune disease, Biomarker, Therapy, Autoimmunity, Humoral immunity, Autoimmune diseases

## Abstract

B cells play a pivotal role in the pathogenesis of autoimmune diseases. Although previous studies have shown many genetic polymorphisms associated with B-cell activation in patients with various autoimmune disorders, progress in epigenetic research has revealed new mechanisms leading to B-cell hyperactivation. Epigenetic mechanisms, including those involving histone modifications, DNA methylation, and noncoding RNAs, regulate B-cell responses, and their dysregulation can contribute to the pathogenesis of autoimmune diseases. Patients with autoimmune diseases show epigenetic alterations that lead to the initiation and perpetuation of autoimmune inflammation. Moreover, many clinical and animal model studies have shown the promising potential of epigenetic therapies for patients. In this review, we present an up-to-date overview of epigenetic mechanisms with a focus on their roles in regulating functional B-cell subsets. Furthermore, we discuss epigenetic dysregulation in B cells and highlight its contribution to the development of autoimmune diseases. Based on clinical and preclinical evidence, we discuss novel epigenetic biomarkers and therapies for patients with autoimmune disorders.

## Introduction

Immune dysregulation contributes to the breakdown of immune tolerance, leading to autoimmune pathogenesis. In recent decades, numerous studies have revealed the significant roles of both innate and adaptive immune cells, especially B cells, in the development of autoimmune diseases [1–3]. The presence of autoantibodies and hyperactivation of B cells have been characterized as salient features in patients with various autoimmune diseases, such as systemic lupus erythematosus (SLE), primary Sjögren’s syndrome (pSS), and rheumatoid arthritis (RA), which highlights a pivotal role of B cells in the pathogenesis of autoimmune diseases [4, 5]. It has been recognized that B-cell dysregulation is critically involved in the initiation and perpetuation of autoimmunity. However, the mechanisms and consequences of B-cell tolerance breakdown in the pathogenesis of autoimmune diseases are still not fully understood.

Genome-wide association studies (GWAS) have identified hundreds of genetic polymorphisms that are associated with increased risks of developing autoimmune diseases, among which many affect genes that modulate the functions of immune cells, including B cells [6, 7]. These autoimmunity-associated risk variants have functions that are highly enriched for cellular processes that regulate B-cell proliferation, differentiation, and activation [8]. However, the low concordance rates of many autoimmune diseases in monozygotic twins suggest the roles of environmental factors and epigenetic mechanisms in disease development [9]. Increasing evidence shows that epigenetic modifications, which regulate gene expression without affecting DNA sequence, are critically involved in the breakdown of B-cell tolerance, contributing to autoimmune inflammation and disease progression in patients [9–11]. Moreover, the differentiation and function of B-cell subsets are regulated by diverse epigenetic events, including changes in histone modifications, DNA methylation profiles, and noncoding RNAs. Epigenetic dysregulation may lead to aberrant expansion of pathogenic B-cell subsets in the development of autoimmune diseases [12, 13]. Genome-wide epigenomic analysis has identified significant alterations in the epigenetic profiles of B cells from patients with autoimmune diseases [14]. Recent studies have revealed that epigenetic signatures, including DNA methylation profiles, may serve as useful biomarkers that can reflect disease prognosis, severity, and responses to therapies [15, 16]. Moreover, clinical investigations and animal studies suggest that targeting epigenetic modifiers could be a novel effective therapeutic strategy [10]. Therefore, the characterization of global epigenetic profiles by high-throughput technologies may facilitate the development of personalized medicine for treating patients. In this review, we discuss epigenetic modifications in different functional B-cell subsets and their roles in disease pathogenesis and clinical therapies for patients with autoimmune diseases.

## Epigenetic modifications regulate B-cell differentiation

### Epigenetic mechanisms

**Fig. 1:**
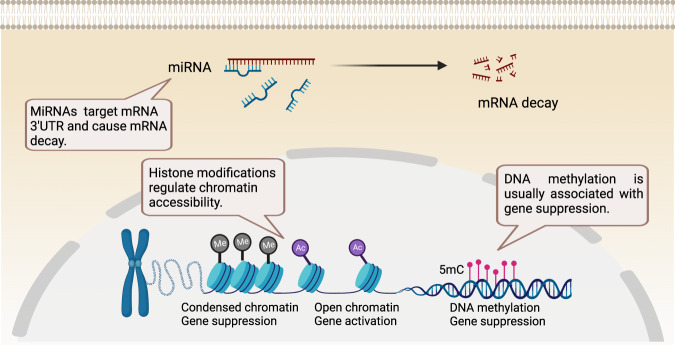
Major epigenetic mechanisms. Histone modifications and DNA methylation affect transcriptional accessibility. MiRNAs bind to target mRNAs and lead to the cleavage and degradation of the mRNAs

In the nucleus, DNA is packaged into chromatin through interactions with histones and many other proteins. Epigenetic changes, such as cytosine methylation in DNA and posttranslational modification of histone amino acid residues, strongly affect chromatin relaxation and condensation, which are closely associated with transcriptional activity. Moreover, epigenetic modifications can directly influence the binding of transcription factors to gene promoters and enhancers. Noncoding RNAs, including long noncoding RNAs (lncRNAs) and microRNAs (miRNAs), are actively involved in the regulation of chromatin structure, gene silencing, and posttranscriptional events. Epigenetic control of RNAs via factors such as N^6^-methyladenosine (m6A) has been shown to play important roles in regulating RNA splicing, translation, and stability. m6A is initially installed by various methyltransferase complexes (“writers”), including methyltransferase-like protein 3 (METTL3), and removed by m6A demethylases (“erasers”), such as fat-mass and obesity-associated protein and alkylation repair homolog protein 5. The effects of m6A are largely dependent on m6A-binding proteins (“readers”), such as YT521-B homology (YTH) domain family members [17]. These epigenetic modifications are important regulators during B-cell differentiation and activation (Fig. 1).

#### Histone posttranslational modifications

As the basic structural unit of the chromosome, the nucleosome is composed of a 147 bp segment of DNA that is wrapped around an octamer of core histone proteins consisting of two copies of each of the histones H2A, H2B, H3, and H4. Histones can be modified at N-terminal residues through various histone-modifying enzymes. These modifications regulate gene expression by changing chromatin structure and accessibility to transcription complexes. Many histone modifications have been reported, and histone acetylation and methylation are most commonly described in the regulation of B-cell functions and autoimmune pathogenesis. Histone acetylation is usually associated with an open chromatin structure and easy binding of transcriptional machinery to transcription sites with increased gene expression. Histone methylation can either promote or suppress gene transcription. Trimethylation at the Lys4 residue of histone H3 (H3K4me3) activates gene transcription and is usually enriched in active promoters around transcription start sites (TSSs) [18, 19]. However, trimethylation at Lys9 and Lys27 of histone H3 (H3K9me3 and H3K27me3, respectively) can function as a silencer to repress gene expression by modifying chromatin architecture and interactions [20, 21]. Other histone modifications, including ubiquitination, phosphorylation, and sumoylation, also directly influence interactions among histones, DNA, and transcription complexes [22, 23].

Histone acetylation is catalyzed by histone acetyltransferases (HATs) and histone deacetylases (HDACs) that can add or remove acetyl groups, respectively. HAT family members have conserved protein–protein interaction and substrate-specific binding domains that can recognize particular genomic sites [24]. Both HATs and HDACs are important regulators during B-cell development and autoimmune diseases. Although HDACs and HATs show contrary functions in histone modification, they are counterbalanced during B-cell development and show complex roles in B-cell homeostasis.

#### DNA methylation

Methylation of the 5’ position of cytosines (5mC), in the context of CpG dinucleotides, is the main form of DNA methylation in mammals and is usually associated with gene suppression. Recently, DNA N^6^-methyldeoxyadenine (6mA) has also been identified in the human genome [25, 26]. These epigenetic modifications in DNA are usually stable and heritable. In the mammalian genome, 5mC can be found in isolated CpG sites or in the context of CpG islands, and its addition is catalyzed by various DNA methyltransferases (DNMTs) with distinct capacities. DNMT1 plays a dominant role in the maintenance of the DNA methylation profiles during cell division, while DNMT3A and DNMT3B are responsible for adding new methyl groups to unmethylated DNA [27]. Apart from their roles in establishing and maintaining DNA methylation patterns, DNMTs have been reported to serve as versatile tools for epigenetic regulation [27, 28]. Recent evidence has indicated diverse functions of DNMTs, including transcriptional silencing, transcriptional activation, and posttranscriptional regulation [27]. Active DNA demethylation is a multistep process that starts with the participation of enzymes of the ten-eleven translocation (TET) family. TET enzymes oxidize methylcytosine to hydroxymethylcytosine, formylcytosine, and carboxycytosine, which are then excised through the activity of thymine DNA glycosylase, followed by base excision repair. TETs and DNMTs cooperate to sustain homeostasis of gene transcription with site-specific dependency [29].

In prokaryotes and protists, DNA 6mA modification is frequently detected. Recent developments in 6mA detection techniques have revealed the presence of 6mA in the genomic DNA of eukaryotes, including mammals [25, 26]. Unlike 5mC, which increases DNA helix stability, 6mA destabilizes the helix and induces DNA unwinding [30]. 6mA modification is a reversible process mediated by 6mA-specific methyltransferases and demethylases. Many studies have shown diverse biological and pathological roles of 6mA in regulating gene transcription, chromatin, and tumor progression [25, 26]. Available data indicate that 6mA may have a conserved role in recognizing and eliminating foreign DNA and thus participate in immune modulation [25]. Alkylation repair homolog 1 (ALKBH1) is a demethylase that regulates 6mA turnover in unpaired regions associated with dynamic chromosome regulation [31]. A recent study showed that ALKBH1 regulates the microenvironment in the bone marrow, where B cells are generated [32], suggesting that ALKBH1 may indirectly regulate B-cell homeostasis. Current evidence on the roles of 6mA in regulating B-cell responses is still limited, and further investigations are needed.

#### Noncoding RNAs

The pervasive transcription of DNA yields the production of numerous noncoding RNAs, including lncRNAs, miRNAs, and circular RNAs. These noncoding RNAs do not encode functional proteins, but they exert diverse functions in regulating gene expression. LncRNAs are generally over 200 nt in length, while miRNAs are much smaller, with 21–25 nucleotides. LncRNAs act as important regulators of chromatin remodeling, gene transcription, and posttranscriptional modification [33]. LncRNAs interact with a variety of targets, including proteins and RNAs, via allosteric effects. Moreover, they can serve as a molecular scaffold to recruit target chromatin-modifying proteins [34]. Unlike lncRNAs, miRNAs generally target mRNAs in the 3′ untranslated region (3′UTR), which leads to the cleavage and degradation of mRNAs and inhibits protein translation. The biogenesis of miRNAs is directly regulated by DNA methylation [35]. MiRNAs also target various DNMTs and thus regulate DNA methylation [36], indicating crosstalk between different epigenetic events.

Emerging evidence suggests that noncoding RNAs play an important role in immune homeostasis and the etiology of human diseases, including autoimmune diseases [37]. It has been shown that various noncoding RNAs sustain B-cell lineage-specific gene expression profiles and are involved in B-cell-related diseases [38]. The aberrant expression profiles of several noncoding RNAs, including miR-150, miR-155, and small nucleolar RNA host gene 14, are associated with B-cell malignancies and autoimmune diseases [39, 40].

#### N6-methyladenosine RNA methylation

m6A is a dynamic and reversible posttranscriptional epigenetic modification that is the most prevalent type of mRNA methylation in eukaryotes. METTL3 and METTL14 are two important methyltransferases responsible for m6A. Deficiency of METTL14 reduces mRNA m6A methylation in B cells and results in severely inhibited proliferation of pro-B cells with severely abnormal gene expression profiles associated with B-cell development, suggesting that m6A mRNA methylation plays an essential role in B-cell development in bone marrow [41]. Consistently, the cytoplasmic m6A reader YT521-B homology domain family member 2 sustains IL-7-induced pro-B-cell proliferation by suppressing a group of transcripts [41]. METTL3-dependent m6A mRNA methylation controls early B-cell differentiation from hematopoietic stem cells in bone marrow [42].

### Epigenetic modifications in functional B-cell subsets

**Fig. 2:**
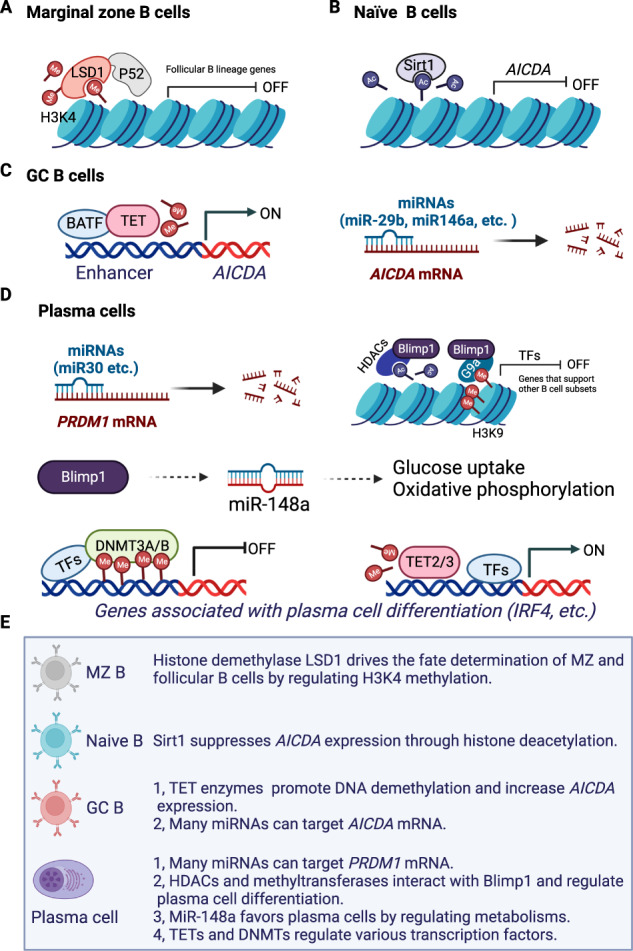
Key epigenetic events in B-cell subsets. **A** The histone demethylase LSD1 controls marginal zone B-cell and follicular B-cell fate. **B** Naive B cells express high levels of Sirt1, which suppresses *AICDA* expression. **C** In germinal center (GC) B cells, TET proteins promote *AICDA* gene transcription through DNA demethylation. *AICDA* mRNA is targeted by various miRNAs. **D** In plasma cells, *PRDM1* mRNA is targeted by miRNAs. Blimp1 suppresses several transcription factors that support other B-cell subsets by interacting with epigenetic modifiers. MiR-148a is regulated by Blimp1 and serves as a key link between Blimp1 and metabolic regulation in plasma cells. DNMT3A/B and TET2/3 sustain the DNA methylation balance and regulate the transcription of genes associated with plasma cell differentiation. **E** Summary of key epigenetic regulatory events in B-cell subsets

During ontogeny, B cells differentiate from hematopoietic stem cells and undergo an ordered maturation and selection process in the bone marrow. Many studies have demonstrated that epigenetic modifications, including those related to DNA methylation, histone modifications, noncoding RNAs, and m6A mRNA methylation, play an important role during the B-cell developmental process in the bone marrow [38]. Newly formed immature B cells migrate from the bone marrow and differentiate into various mature functional B-cell subsets, including marginal zone (MZ) B cells, germinal center (GC) B cells, plasma cells, memory B cells, and regulatory B (Breg) cells, in the peripheral lymphoid organs. These B-cell subsets exhibit diverse functions, including antigen presentation, antibody secretion, and cytokine production, under different conditions. Maturation and activation of peripheral B cells are mainly dependent on environmental factors such as B-cell activating factor (BAFF), ligands of Toll-like receptors (TLRs), and IL-21. Recent studies have highlighted the essential roles of epigenetic regulation in different B-cell subsets (Fig. 2), and this epigenetic regulation contributes to the pathogenesis of autoimmune diseases, including SLE, pSS, and RA [5, 8, 43, 44].

#### Marginal zone B cells

Immature B cells migrate from the bone marrow and enter the spleen as transitional B cells, where they encounter survival signals, including BAFF, in the tissue microenvironment and complete their fate decision as either MZ B cells or follicular B cells. MZ B cells are located at the borders of red pulp and white pulp. Early studies showed that MZ B cells are innate-like lymphocytes and are important for housekeeping functions such as the clearance of apoptotic cell debris. MZ B cells express B-cell receptors (BCRs) with polyreactivity and respond to various TLR stimulations, which drives the rapid production of low-affinity antibodies against both T-cell-independent and T-cell-dependent antigens [45, 46]. MZ B cells are characterized by low expression levels of surface IgD and high expression levels of IgM and CD21/CD35.

Expanded MZ B-cell populations that produce massive levels of autoantibodies are detected in some patients with autoimmune diseases [45]. However, autoimmune vasculitis patients show reduced frequencies and numbers of circulating MZ-like B cells, suggesting complex roles of MZ B cells in autoimmune disorders [47]. Although MZ B cells undergo clonal expansion with aberrant autoantibody production and can enter lymphoid follicles to interact with CD4 T cells in lupus-prone mice [48, 49], a recent study showed defective MZ B-cell differentiation in SLE patients [50]. Reduced MZ B-cell frequency is observed in SLE patients, and the reduction is even more prominent in those with lupus nephritis, which might be associated with the reduction of their putative transitional B-cell precursors [50]. Recently, it has been reported that MZ B-cell differentiation is regulated by lysine-specific demethylase 1 (LSD1), a histone demethylase that targets H3K4me1, H3K4me2, H3K9me1, and H3K9me2 through flavin adenine dinucleotide-dependent amine oxidation [51, 52]. Conditional deletion of LSD1 in B cells results in a dramatic reduction in MZ B cells, while follicular B cells are not affected. Moreover, LSD1 regulates chromatin accessibility at the motifs of several key transcription factors for B-cell development and thus sustains the transcriptional identity of MZ B cells [51]. Mechanistically, LSD1 acts as an epigenetic regulator and cooperates with p52, a subunit of NF-κB with DNA binding abilities, to drive the fate determination of MZ and follicular B cells (Fig. 2A) [51].

#### Germinal center B cells

Upon proper antigen stimulation, naive B cells undergo rapid proliferation with the help of cognate CD4 T cells within lymphoid follicles and form GCs, a specialized microanatomical structure that supports cell division, somatic hypermutation (SHM), and class switch recombination (CSR) of activated B cells. GC B cells are characterized by surface phenotypic markers of peanut agglutinin (PNA)^+^, Fas^+^, and IgD^lo^. In lymphoid tissues, GC structures can be divided into two major areas: the light zone and the dark zone. GC B cells undergo intensive proliferation in the dark zone, where activation-induced cytidine deaminase (AID) critically regulates SHM at the variable regions of immunoglobulin genes to increase BCR affinities. In the light zone, B cells expressing BCRs with high affinity for the antigens are selected. The bidirectional movement of GC B cells in the light zone and dark zone results in iterative rounds of SHM and selection, which ultimately generates B cells with high affinity for the antigen.

Compared with naive B cells, GC B cells are predominantly hypomethylated and show dramatic reorganization of the genomic architecture with massive unpacking of chromosomes [53, 54]. *Mettl3*-deficient GC B cells exhibit reduced cell cycle progression and decreased expression of proliferation- and oxidative phosphorylation-related genes, suggesting that m6A modifications by METTL3 are required for GC maintenance [55]. GC B cells exhibit a unique transcriptional network. GC B cells are identified by the high expression levels of several key transcription factors. B-cell lymphoma 6 (BCL6) is a key transcription factor for GC B cells. The epigenetic regulation of BCL6 plays a critical role during the GC reaction. Deletion of a GC-specific region upstream of *Bcl6* that shows frequent intrachromosomal interactions results in diminished GC formation without affecting other developmental stages [53]. Furthermore, dysregulated epigenetic regulation of BCL6 in B cells is associated with GC-derived lymphoma [56]. T follicular helper (Tfh) cells, characterized by high expression of BCL6, play an essential role in the GC reaction and autoimmune pathogenesis [3]. Ubiquitin-like with PHD and RING finger domains 1 (UHRF1) suppresses DNA methylation and decreases the level of H3K27m3 within BCL6 promoter regions and thus controls Tfh cell differentiation and the GC reaction. Decreased expression of UHRF1 results in abnormal Tfh expansion and GC responses, which promotes the development of SLE [57].

GC B cells express high levels of AID. As an essential step in SHM and CSR of immunoglobulin genes, AID induces the conversion of deoxycytidine to deoxyuracil and drives base pair mismatches in DNA. The recruitment of AID at IgH switch regions is regulated by the histone methyltransferase multiple myeloma SET domain, the RNA exosome cofactor MPP6 and the nuclear m6A-modified RNA reader YTH domain containing 1, suggesting epigenetic regulation of AID-mediated DNA breaks during CSR [58, 59]. Although AID mainly binds to the Ig gene locus, chromatin immunoprecipitation analysis shows a genome-wide presence of AID in activated B cells [60], suggesting diverse functions of AID. In addition to inducing base pair mismatch, AID also shows epigenetic regulatory functions during the GC reaction. Although GC B cells show marked locus-specific loss of DNA methylation, deficiency of AID results in abrogated CpG hypomethylation during the GC reaction [61]. Moreover, AID also contributes to the epigenetic diversity of GC B cells [61, 62]. Overexpression of AID increases cytosine methylation heterogeneity, whereas AICDA depletion leads to reduced heterogeneity characteristic of normal GC B cells [62]. These epigenetic effects of AID are dependent on TET2, as AID-mediated demethylation is markedly impaired in *Tet2*-deficient GC B cells [63]. Interestingly, AID might be involved in the establishment of DNA methylation patterns even before the GC reaction [64]. AID induces DNA breaks and drives CSR in GC B cells.

AID expression can hardly be detected in resting B cells but is strongly induced by T-cell-dependent and T-cell-independent antigenic stimuli and effector molecules, including CD40L, TLR ligands, and cytokines. The induction of *Aicda*, which encodes AID, is regulated by epigenetic modifications, including histone acetylation and DNA demethylation around the gene locus [65, 66]. B-cell-specific deficiency of HDAC3 results in an impaired GC reaction with a reduction in dark zone centroblasts and accumulation of light zone centrocytes [67]. The NAD^+^-dependent class III HDAC sirtuin 1 (Sirt1), a nonclassical class III HDAC, is highly expressed in resting B cells but downregulated upon B-cell activation (Fig. 2B). Deficiency of *Sirt1* results in increased *Aicda* expression through acetylation of *Aicda* promoter histone and nonhistone proteins, suggesting that Sirt1 acts as a key suppressive epigenetic regulator of *Aicda* during B-cell activation [66]. DNA methylation and demethylation markedly affect the GC B-cell reaction (Fig. 2C). Deficiency of *Tet2* and *Tet3* promotes GC B-cell responses, while deletion of *Dnmt1* abrogates this effect [68]. Moreover, combined *Tet2* and *Tet3* loss-of-function in GC B cells increases C-to-T and G-to-A transition mutagenesis, suggesting that TET enzymes may affect SHM [69]. Indeed, TET enzymes promote DNA demethylation and maintain chromatin accessibility at the superenhancer region of the *Aicda* locus [65]. Furthermore, basic leucine zipper transcription factor ATF-like (BATF) plays a key role in TET-mediated *Aicda* expression [65]. The short-chain fatty acids (SCFAs) butyrate and propionate are common metabolites with diverse immunomodulatory functions. It was recently found that SCFAs inhibit *Aicda* expression in a dose-dependent manner by increasing the levels of certain miRNAs that target the Aicda 3′-UTR through inhibition of HDACs targeting miRNA host genes [70]. The transcription of *Aicda* is also regulated by noncoding RNAs such as miR-29b and miR146a (Fig. 2C) [71, 72]. Moreover, m6A modifications by METTL3 are essential for maintaining GC responses in peripheral lymphoid organs [55]. METTL3 enzyme-catalyzed m6A in IgH locus-associated G-rich long noncoding RNA (SμGLT) drives recognition and 3’-end processing by RNA exosomes, which suppresses chromosomal translocation and CSR [59]. METTL3 also suppresses IgH-associated abnormal DNA breaks and improves genomic stability [59]. The RNA exosome complex is a critical regulator of noncoding RNAs. DIS3, an important RNase subunit within RNA exosomes, was recently found to regulate chromosomal architecture and SHM in B cells [73]. Ablation of DIS3 results in insufficient noncoding RNA turnover and accumulation of DNA‒RNA hybrids, which affects somatic mutation at the *Igh* locus [73]. Overexpression of miR-29b in human B cells decreases AID expression and impairs CSR to IgE [71]. TGF-β imitates CSR to IgA by activating Smad2, Smad3, and Smad4. miR146a is abundantly expressed in resting B cells, whereas activated B cells undergoing CSR show diminished miR146a levels [74]. Deletion of *miR146a* leads to increased levels of Smad2, Smad3, and Smad4 and promotes CSR to IgA, suggesting that miR146a may regulate CSR by targeting TGF-β signaling [74].

#### Plasma cells

Plasma cells are terminally differentiated B cells with potent antibody-secreting abilities that are generated either from follicular GC reactions or from activated extrafollicular B cells. GC B-cell-derived plasma cells produce class-switched antibodies with high affinity and have a long lifespan, whereas extrafollicular B-cell-derived plasma cells are short-lived cells that mainly produce IgM with low affinity. Numerous studies have shown the diverse functions of plasma cells in infections, cancers, and autoimmune diseases [43, 75]. Plasma cells exhibit unique phenotypic, transcriptional, metabolic, and functional characteristics. The differentiation of antibody-secreting plasma cells is a coordinated process with both genetic and epigenetic regulation [76].

Plasma cells show high expression levels of B-lymphocyte-induced maturation protein 1 (Blimp1), which plays a central role during plasma cell differentiation. The mRNA of the *Prdm1* gene that encodes Blimp1 has a long 3’-UTR and is targeted by many miRNAs, such as miR30 family members (Fig. 2D) [77, 78]. These miRNAs directly target *Prdm1* mRNA, leading to suppressed Blimp1 expression and inhibited plasma cell differentiation [78]. Blimp1 suppresses several transcription factors that support other B-cell subsets, such as BCL6, through diverse epigenetic modifications (Fig. 2D). Blimp1 interacts with HDACs, regulates histone acetylation and thus suppresses the expression of target genes such as *Bcl6* and *cMyc* [77, 79]. Moreover, Blimp1 recruits methyltransferases and promotes H3K9me3 deposition in the promoter regions of *Pax5* and *Spib* [77, 80].

DNA methylation not only is important for GC B-cell differentiation but also plays a key role in sustaining plasma cell identity (Fig. 2D). Genetic deletion of DNMT3 promotes the expansion of GC B cells and plasma cells. Gene expression is mostly normal in naive and GC B cells but is prominently dysregulated in Dnmt3-deficient plasma cells [81]. In particular, dysregulated DNMT3-dependent DNA methylation coincides with E2A and PU.1-interferon regulatory factor (IRF) composite-binding motifs in plasma cells, suggesting that de novo DNA methylation by DNMT3 inhibits plasma cell differentiation by repressing the gene expression program of key B-cell fate and activation genes [81]. In contrast, DNA demethylation mediated by TET2 and TET3 is essential for plasma cell differentiation since conditional Tet2/3 double-KO B cells fail to differentiate into plasma cells upon antigen immunization [82]. TET2- and TET3-dependent demethylation is dispensable for initial IRF4 expression but is essential for sustaining high IRF4 expression, which is required for plasma cell differentiation [82]. Interestingly, ascorbic acid, an essential vitamin for humans, promotes plasma cell differentiation and humoral responses by increasing TET2/3-mediated DNA demethylation [83], supporting an important role of DNA demethylation during plasma B-cell differentiation.

Many miRNAs regulate the differentiation of plasma cells through diverse effector mechanisms. MiR-148a is abundantly expressed in plasma cells. B-cell-specific deficiency of miR-148a results in reduced numbers of antibody-secreting plasma cells. Moreover, miR-148a promotes plasmablast differentiation from GC B cells in mice. Transcriptome and metabolic analyses suggest that miR-148a favors plasma cells by fine tuning glucose uptake and oxidative phosphorylation [84]. Longevity is a hallmark of long-lived plasma cells derived from GC B cells. Many miRNAs, including miR-155, are important for sustaining plasma cell survival [85]. Recently, it was found that miR-29 controls apoptosis of mature B cells via the phosphatase and tensin homolog deleted on chromosome 10 (PTEN)-phosphoinositide 3-kinase (PI3K) axis signaling pathway, suggesting that miR-29 may play a role in sustaining plasma cell survival [86].

One of the most prominent biological features of plasma cells is their unique metabolic pattern that sustains the massive production of antibodies. Plasma cells primarily rely on oxidative phosphorylation to support the energy demand for producing antibodies, whereas GC B cells utilize fatty acid oxidation more than glycolysis and oxidative phosphorylation [87, 88]. The longevity of plasma cells is also dictated by metabolic pathways [89, 90]. The import of pyruvate into mitochondria is important, as the loss of mitochondrial pyruvate carriers leads to a progressive decrease in long-lived plasma cells in mice [91]. Plasma cells also require a high rate of glucose uptake for glycolysis and antibody glycosylation [92]. The metabolic signature is largely dependent on Blimp1, the key transcription factor of plasma cells [76]. Interestingly, miR-148a is regulated by Blimp1 and serves as a key link between Blimp1 and metabolic regulation in plasma cells (Fig. 2D) [84]. Blimp1-induced miR-148a controls energy metabolism in plasma cells by regulating Glut-1-mediated glucose uptake and mitochondrial respiration [84]. Enhancer of zest 2 (EZH2) is the catalytic subunit of polycomb repressive complex 2 (PRC2), which mediates H3K27 trimethylation. EZH2 directly interacts with Bcl6 and controls GC formation in mice [93]. Moreover, EZH2 is required for the metabolic programming of plasma cells. Deficiency of *Ezh2* results in impaired plasma cell differentiation accompanied by reduced glycolysis and oxidative phosphorylation [94].

#### Memory B cells

Memory B cells are important for rapid immune responses against repeated infection. The effectiveness of vaccines against infectious pathogens is largely dependent on the formation of memory B cells [95, 96]. Both memory B cells and plasma cells can be derived from GC-dependent and GC-independent pathways, but these two B-cell populations show distinct transcriptional, phenotypic, and functional features [97]. Unlike plasma cells, memory B cells are normally in a resting state and can mount a strong and rapid response to a second challenge [98]. The terminal differentiation of GC B cells into plasma cells versus memory B cells is largely regulated by the extent of signals received from BCR engagement and T-cell help. BTB domain and CNC homology 2 (BACH2) is a transcription factor that represses the expression of plasma cell-defining factors, including Blimp1. GC B cells with weak T-cell help do not transduce sufficient NF-κB signals to repress BACH2 and have a strong potential to differentiate into memory B cells [97]. In contrast, epigenetic suppression of BACH2 by *EZH2* promotes plasma cell but not memory B-cell differentiation [99]. Integrative analysis of transcriptome factors, including mRNAs, miRNAs, lncRNAs, chromatin accessibility, and cis-regulatory elements, revealed a core mRNA-noncoding RNA transcriptional signature of human memory B cells [100]. Memory B cells have an accessible chromatin architecture around several plasma cell-specific genes, including *Xbp1*, *Prdm1*, and *Irf4*, suggesting the reactivation potential of memory B cells [101].

Memory B cells are heterogeneous and have various subsets. Previous studies identified different memory B-cell subsets in mice based on the expression of CD80, CD86, PDL2, and BCR isotype [97, 98]. Recently, atypical memory B cells were identified to be characterized by high expression of CD11c and T-bet and low expression of CD27 and CD21 [5, 98, 102]. Increasing evidence shows the critical involvement of atypical memory B cells during infections and autoimmune diseases [103, 104]. Expansion of CD11c^+^ atypical memory B cells is observed in the peripheral blood of SLE patients. Moreover, CD11c^+^ B cells from SLE patients can differentiate into autoantibody-producing plasma cells upon IL-21 stimulation in culture [104, 105]. It has been well recognized that the lncRNA Xist can regulate X-chromosome inactivation (XCI). Epigenetic profiling shows that Xist is lost from the inactivated chromosome at the pro-B-cell stage but is restored upon B-cell activation, suggesting that Xist RNA localization is important for regulating XCI during B-cell development and activation in females [106]. Conventional memory B cells show dispersed Xist RNA signals across the genome, while atypical memory B cells exhibit distinct Xist RNA localization patterns [107]. The loss of Xist promotes the differentiation of CD11c^+^ B cells, suggesting that Xist-mediated XCI maintenance may contribute to the generation of CD11c^+^ atypical memory B cells during SLE development [13].

#### Regulatory B cells

Breg cells represent a special B-cell subpopulation with potent immunosuppressive functions. Bregs maintain immune tolerance by suppressing various inflammatory populations, including Th17 cells, Th1 cells, CD8 T cells, monocytes, and dendritic cells (DCs) [108]. Bregs produce many suppressive cytokines, such as IL-10, TGF-β, and IL-35 [108]. Bregs are heterogeneous and have various phenotypes, including CD5^+^CD1d^hi^ B cells, Tim-1^+^ B cells, and LAG-3^+^CD138^hi^ plasma cells [108, 109]. Currently, the developmental origin of Bregs is unclear. Immature B cells, MZ B cells, and plasma cells all have the potential to differentiate into IL-10-producing B cells in the presence of BAFF, lipopolysaccharide (LPS), CD40L, IL-21 or other stimuli [108, 110, 111], suggesting that environmental factors and not lineage specificity determine the generation of Bregs. Numerous studies have demonstrated an important role of Bregs in infection, inflammation, cancer, and autoimmunity [44, 110, 112–115]. Adoptive transfer of Bregs suppresses T-cell responses and ameliorates disease development in mice with experimental Sjögren’s syndrome, collagen-induced arthritis (CIA), and SLE, suggesting therapeutic potential of Bregs in autoimmune diseases [44, 116, 117].

Although it is unclear whether Breg identity is determined by a specific transcription factor, increasing evidence shows a pivotal role of epigenetic regulation in Bregs. IL-10 is a key effector cytokine for the suppressive functions of Bregs. DNA methylation profiling has shown that IL-10-producing B cells are characterized by a specific methylation signature around the *IL10* TSS, suggesting an important role of DNA methylation in regulating IL-10 production by B cells [118]. Moreover, histone modifications are also involved in the induction of IL-10-producing B cells. Inhibition of HDAC11 increases IL-10 production by B cells in patients with allergic rhinitis [119]. Entinostat, an HDAC inhibitor, inhibits the binding of HDAC1 to the promoter region of *Il10* and promotes the induction of IL-10-producing B cells by LPS [120]. Another HDAC inhibitor, trichostatin A, increases the frequency of IL‑10- and TGF‑β‑producing CD5^+^CD1d^high^ B cells in vitro and in vivo [121], suggesting that HDAC inhibitors might be potential agents for treating autoimmune diseases and transplant rejection. SCFAs from the environment not only epigenetically regulate the generation of GC B cells and plasma cells but also promote IL-10 expression dependent on their HDAC inhibitory activity [70, 122].

#### B1 cells

B cells are divided into two major lineages: B1 cells and B2 cells. B2 cells are conventional B cells that can be activated and produce antigen-specific antibodies. B1 cells are long-lived self-renewing cells that spontaneously produce natural antibodies. B1 cells are mostly located in peritoneal and pleural cavities and exhibit innate-like features. B1 cells are characterized by surface expression of CD45RA, CD11b, and CD43. Based on the expression levels of CD5, B1 cells are further divided into B1a (CD5^+^) and B1b (CD5^-^) subsets [123, 124]. B1 and B2 cells also show different transcriptomes, metabolic statuses, and epigenetic modifications [123, 125].

Genome-wide CpG methylation analysis has revealed that B1a cell development is characterized by programmed demethylation at enhancers that are methylated in B2 cells, which is associated with B1 lineage-specific gene expression [125]. Interestingly, B-cell-specific DNMT3a deficiency results in the selective expansion of B1a cells, suggesting that DNMT3a-dependent CpG methylation may control B-cell lineage-specific gene expression [125]. Inhibition of HDAC activity promotes the migration and function of B1 cells, suggesting an important role of histone acetylation in regulating B1-cell functions [126]. Since B1 cells require Blimp1 for antibody secretion in early protection against pathogens [127, 128], the epigenetic modification of *Prdm1* may be important for sustaining the normal functions of B1 cells. CD5 is an important marker of B1a cells. It has been reported that CD5 expression in B cells from SLE patients can be regulated by IL-6-induced DNA methylation, indicating a potential role of cytokines in regulating B-cell phenotypes [129].

## Epigenetic dysregulation of B cells in autoimmune diseases

During the past decades, extensive evidence from clinical investigations and animal studies has demonstrated the significance of B-cell hyperactivation during the development of autoimmune diseases. B-cell activation and differentiation are critically regulated by epigenetic modifications (Fig. 2E). Importantly, successful applications of B-cell-targeted therapies in some autoimmune diseases have highlighted the pathological significance of B cells in autoimmune pathogenesis. Despite recent studies revealing a pivotal role of B cells in autoimmunity, the triggers and subsequent consequences of B-cell tolerance breakdown have not been fully elucidated.

### Dysregulated B-cell signaling in autoimmune diseases

Hyperactivation of B cells with massive production of autoantibodies and cytokines are hallmark features of many autoimmune diseases, including SLE, pSS, and RA. Accumulating evidence suggests that dysregulated B-cell signaling drives autoimmune development by promoting the activation and differentiation of autoreactive B-cell clones [8]. It has been well recognized that the microenvironment is important for B-cell differentiation and activation in the bone marrow and peripheral lymphoid organs. Intrinsic BCR signals, cytokines, and TLR ligands shape B-cell phenotypes and drive autoimmune GC reactions. Consequently, the breakdown of immune tolerance leads to the development of systemic autoimmunity [8].

#### BCR signaling

During B-cell development, BCR signaling controls the negative and positive selection of immature B cells in the bone marrow, which largely shape the BCR repertoire. A sustained BCR signal is essential for the survival of both mature and immature B cells, whereas strong BCR signaling may also promote B-cell apoptosis. Thus, an intermediate BCR intensity is optimal for sustaining B-cell survival. In general, B cells with BCRs that recognize self-antigens are removed during negative selection. However, genetic polymorphisms associated with BCR signaling may affect the generation of autoreactive B cells. A single-nucleotide polymorphism (SNP) in protein tyrosine phosphatase nonreceptor type 22 (PTPN22) (W620) that negatively regulates BCR downstream signaling is associated with increased risks of several autoimmune diseases, including SLE, RA, and type 1 diabetes (T1D), suggesting a fundamental role for BCR signaling in the development of autoimmunity [130–132]. PTPN22 has broad effects on both T-cell and B-cell selection and function. Although it is not clear whether the SNP is a gain-of-function or loss-of-function variant, studies of animal models suggest a gain-of-function for the *PTPN22* variant in the development of autoimmune diseases [8, 133]. Nonobese diabetic (NOD) mice with *Ptpn22* deficiency show reduced numbers of plasma cells, and B-cell-specific ablation of *Ptpn22* decreases the incidence of diabetes in NOD mice, suggesting that *Ptpn22* variation may contribute to T1D by modifying B-cell maturation [8, 133]. Moreover, *PTPN22* (W620) affects the expression of various surface receptors in B cells and may modulate B-cell tolerance via diverse mechanisms [8, 132]. The *PTPN22* (W620) risk allele shows a dominant effect in regulating autoreactive B cells even before the onset of autoimmunity, which might be associated with altered BCR signaling and upregulation of genes that promote B-cell responses such as *CD40, TNF receptor-associated factor 1* (*TRAF1*), and *IRF5* [134].

Bruton’s tyrosine kinase (BTK) is a well-known downstream molecule of BCR signaling. BTK is essential for B-cell survival, proliferation, and function. Spontaneous mutations that lead to insufficient BTK function usually cause a dramatic loss of mature B cells and reduced levels of serum antibodies, resulting in X-linked agammaglobulinemia [135]. Many studies have shown that BTK inhibitors effectively ameliorate disease pathology in various animal models of autoimmune diseases [136, 137]. Administration of evobrutinib, a novel BTK inhibitor, shows robust efficacy in mouse models of RA and SLE, as reflected by significant reductions in disease severity and histological damage [137].

The activated B-cell subtype of diffuse large B-cell lymphoma requires chronic active BCR signaling for survival and is resistant to the BTK inhibitor ibrutinib [138]. This resistance is due to epigenetic rather than genetic changes that circumvent BTK blockade, suggesting the potential roles of epigenetic regulation in BCR-driven B-cell hyperactivation [138]. Several clinical trials have shown promising results of BTK inhibitors for treating patients with various autoimmune diseases, such as RA and multiple sclerosis (MS) [136].

The diversity of the BCR repertoire is an important factor that modulates the development of autoimmune diseases. Single-cell RNA sequencing (scRNA-seq) and high-throughput sequencing technologies have made significant contributions to the systemic analysis of the BCR repertoire. Compared with those from healthy donors, B cells from SLE patients show increased BCR clonotypes and biased usage of BCR V(D)J genes [139]. In particular, B cells from SLE patients who are sensitive to immunosuppressive drugs show significantly decreased BCR expression and clonal diversification, while these changes are undetectable in nonsensitive lupus patients, indicating that alterations of the BCR repertoire are associated with sensitivity to immunosuppressive therapy [140]. It has also been reported that the nonresponse rates to rituximab among RA patients are closely associated with marked disruption of the BCR repertoire, suggesting that BCR clonality may serve as a predictor of the responses of RA patients to B-cell-depletion therapy [141]. Although the mechanisms underlying the establishment and changes of the BCR repertoire in autoimmunity are still not fully understood, further studies will shed light on the generation of autoreactive B cells, which will facilitate the development of personalized medicine for the treatment of autoimmune diseases.

#### CD40

During an adaptive immune response, cognate T cells help sustain B-cell survival and promote B-cell differentiation. CD40 is a TNF receptor superfamily member widely expressed in various immune and nonimmune cell populations. The interaction between CD40 expressed on B cells and its binding ligand CD40L (CD154) on CD4 T cells plays an important role during the GC reaction and B-cell differentiation into plasma cells. Many studies have reported aberrant CD40 signaling in patients with autoimmune diseases, which supports the notion that the CD40-CD40L interaction contributes to B-cell hyperactivation and the maintenance of autoimmunity [142]. Blockade of CD40L has been shown to inhibit autoantibody production and tissue inflammation in mice with autoimmune thyroid diseases and NOD mice with SS-like phenotypes, suggesting a critical role of the CD40-CD40L interaction in these diseases [143]. Many biological agents targeting CD40 signaling have been evaluated in clinical trials for their therapeutic efficacy in patients with autoimmune diseases and have achieved partially satisfactory outcomes [142].

#### TLRs

Apart from T-cell-dependent stimulation, B cells can also be activated independent of T cells in response to pathogen-associated molecular patterns (PAMPs) and damage-associated molecular patterns (DAMPs) by TLRs. Bacterial and viral-derived PAMPs such as LPS, RNA, and DNA can be recognized by surface and cytosolic TLRs, which directly activate B cells even without BCR engagement. Moreover, B cells can also be activated by self-derived DAMPs, including double-stranded DNA (dsDNA), which may drive the development of autoimmunity [144]. During B-cell development, combined BCR and TLR signals orchestrate the selection of self-reactive B cells in peripheral organs [8]. Myeloid differentiation factor 88 (MyD88) and interleukin-1 receptor-associated kinase 4 (IRAK-4) are important adaptors of most TLR signaling pathways. Patients with a deficiency of MyD88 or IRAK-4 exhibit defective central and peripheral B-cell tolerance checkpoints and accumulation of autoreactive naive B cells in blood [145]. In patients with Wiskott–Aldrich syndrome, an X-linked immunodeficiency disease frequently associated with systemic autoimmunity, modest alterations in BCR and TLR signaling can disrupt B-cell tolerance by regulating the positive selection of self-reactive B cells [146].

Although human B cells express low levels of TLR4, our previous study identified a novel TLR4^+^CXCR4^+^ plasma cell subset that played a pathogenic role in the pathogenesis of SLE [147]. Blockade of TLR4 significantly decreased serum autoantibody levels and attenuated renal damage in lupus mice, suggesting that TLR4 might be a potential target for prohibiting plasma cell responses and treating SLE [147]. In addition to classic Myd88 signaling, TLR4 signals can also be transduced by BCR and its key adaptor spleen tyrosine kinase in B cells [148]. Genetic association studies have identified TLR polymorphisms as important risk factors for autoimmune diseases, including SLE. In particular, increased expression of TLR7, which recognizes single-stranded RNA, is suggested to be a risk factor for SLE [149]. Interestingly, TLR7 usually escapes interaction with XCI, which may lead to gender bias in SLE patients [150]. Biallelic B cells with higher TLR7 expression exhibit a significantly higher propensity to switch to the IgG class than monoallelic B cells during plasma cell differentiation due to the elevated responsiveness to TLR7 ligands [150]. Evidence from mice with overexpression or deficiency of TLR7 further demonstrates a critical involvement of TLR7 signaling in autoimmunity [8, 149, 151]. Lupus-prone mice with B-cell-specific *Tlr7* deletion show inhibited B-cell responses, decreased levels of class-switched autoantibodies against RNA-associated autoantigens, and diminished systemic autoimmunity [152]. TLR7 signaling in B cells is most prominently associated with GC reactions, leading to systemic autoimmunity [146]. In contrast, TLR9, which interacts with DNA containing unmethylated cytosine-phosphate-guanosine motifs, plays a protective role in many autoimmune diseases. SLE patients show impaired TLR9 but intact TLR7 responses in B cells [153]. B-cell-intrinsic *Tlr9* deficiency results in increased systemic inflammation and immune complex-related glomerulonephritis but decreased anti-nucleosome antibody levels in lupus mice, suggesting that TLR9 regulates lupus development independent of autoantibodies [152, 154]. Deficiency of *Tlr9* in other immune populations, including DCs, plasmacytoid DCs, and neutrophils, has undetectable effects on disease manifestations [154]. Overexpression of TLR9 in B cells ameliorates nephritis in lupus mice, further indicating a protective role of B-cell-intrinsic TLR9 signaling in autoimmunity [154]. However, B-cell-specific deletion of TLR9 also protects mice from T1D development. The protective effects might be associated with increased production of IL-10 by B cells [155]. The functional interaction between TLR7 and TLR9 within B cells is important for B-cell dysregulation in autoimmunity. Unlike TLR4, which is located on the plasma membrane, TLR7 and TLR9 can only recognize internalized ligands in endosomes. Similar pathways participate in the internalization of ligands for TLR7 and TLR9, as both processes require BCR-mediated endocytosis and the formation of endosomes. The antagonistic interaction between TLR7 and TLR9 in B cells might be partially explained by their trafficking to endosomes [149]. Further investigations are needed to elucidate the differential roles of TLRs in B-cell dysregulation and the development of autoimmunity.

#### BAFF

As an important survival factor for B cells, BAFF is considered an important player in systemic autoimmune diseases [156]. BAFF binds to three distinct receptors, namely, BAFFR, transmembrane activator and CAML interactor, and B-cell maturation antigen. Soluble BAFF exists as a 3-mer or in multimers of up to 60, and its different forms may have different binding abilities to the three receptors [156]. BAFF signaling together with BCR pathways orchestrates B-cell development. The interplay between BAFF receptors and BCR promotes B-cell survival and modulates B-cell selection [8]. BAFFR is widely expressed in various B-cell subsets. Although BAFF is essential for maintaining naive B-cell survival, B-cell-intrinsic BAFFR is dispensable for the survival and function of GC B cells but controls GC-independent memory B-cell responses in early immune defense [157]. Memory B cells express BAFFR and require both BCR and BAFFR signaling for their long-term survival [158]. Although BAFF is mostly known as a key B-cell survival factor, it exerts diverse functions in the B-cell response. BAFF promotes IL-10 and IL-35 production by MZ B cells with increased suppressive abilities [156, 159]. BAFF signaling also increases metabolic capacity and regulates redox balance in B cells. BAFF promotes glycolysis and mitochondrial oxidative phosphorylation through the PI3K/Akt pathway, which provides necessary molecular building blocks and energy that support cell mass generation [160].

A variant of *TNFSF13B*, which encodes BAFF, is associated with MS and SLE because it increases soluble BAFF levels and enhances humoral immunity [161]. Indeed, increased levels of BAFF are observed in many patients with autoimmune diseases, including SLE, pSS, and RA [156]. Although it is still controversial, several cross-sectional studies suggest that BAFF serum levels are positively correlated with disease severity in SLE patients [162]. BAFF transgenic mice show several hallmark features of SLE and SS, including the presence of autoantibodies, immune complex-mediated glomerulonephritis, and salivary gland dysfunction and inflammation [8, 163]. The mechanisms by which BAFF promotes autoimmunity are not yet fully understood. Current evidence suggests that excess BAFF levels contribute to the selection of autoreactive B cells and promote the long-term survival of autoantibody-secreting plasma cells [8, 156]. BAFF is mainly produced by myeloid cells such as neutrophils, DCs, and monocytes. Moreover, Tfh cells within GC regions might be an important source of local BAFF that promotes the selection of high-affinity B-cell clones, which may contribute to autoantibody production and systemic autoimmunity [8, 164]. Many biological agents that target BAFF have been developed, and clinical trials suggest promising therapeutic effects in patients with autoimmune diseases such as SLE [156]. Further investigations of strategies targeting different forms of BAFF might be important for the personalized treatment of autoimmune patients.

#### Epigenetic regulation of B-cell hyperactivation

Recent studies have revealed the significant roles of epigenetic regulation during B-cell hyperactivation, which may contribute to immune tolerance breakdown and the development of autoimmunity. B-cell hyperactivation is closely associated with multiple signaling molecules, including BCR, CD40, and TLRs. During GC reactions and plasma cell differentiation, SHM and CSR largely shape the BCR repertoire, which is tightly regulated by many epigenetic events. AID, an important regulator of SHM, increases BCR affinities by diversifying the variable regions of immunoglobulin genes. The transcription and expression of *AICDA* are regulated by histone modifications, DNA methylation, and various miRNAs (Fig. 2) [63, 65, 66, 71, 72], which directly modulate BCR clonotypes during autoimmune disease progression. Epigenetic mechanisms are involved in regulating CD40 expression, as revealed by the finding that HDAC inhibitors can alter the acetylation of histones in chromatin and enhance CD40 expression [165]. Moreover, miRNAs such as miR146a target multiple genes associated with the CD40 pathway and regulate B-cell activation and GC reactions. Although miR146a does not directly target the *CD40* 3’UTR, it can bind to mRNAs of *IKKA, REL*, and *TRAF6*, all of which are important components of the CD40 signaling pathway [72]. MiR146a also indirectly controls CD40 expression by targeting STAT1, which is indispensable for interferon γ-driven CD40 induction [72]. Epigenetic regulation of TLRs is observed in diverse conditions. The lncRNA Xist maintains XCI in normal B cells, whereas Xist dysregulation leads to the escape of X-linked genes such as *TLR7* [150]. Earlier studies suggested that DNA methylation in the human *TLR2* promoter region was associated with suppressed *TLR2* expression in monocytes [166]. Gene transcription of *TLR4* is also downregulated by histone deacetylation and DNA methylation [166]. However, the exact epigenetic regulation mechanisms affecting TLR signaling pathways during B-cell hyperactivation need further investigation. As an important epigenetic regulator, miRNAs can regulate TLR signaling pathways by targeting TLRs and associated adaptor proteins. Many miRNAs, such as miR-155, miR-146, and miR-21, are induced by TLR activation and in turn regulate the expression of TLR pathway components and TLR-induced cytokines [167]. LPS stimulation activates TLR4 signaling and induces the expression of miR-155 in B cells, which is crucial for GC responses and the production of cytokines by B cells [167–169]. The activation and differentiation of B cells also require negative regulators. A recent study observed high expression of Jmjd1c, a member of the JmjC domain-containing histone demethylase family, in B cells but not in other immune cells, which protected mice from autoimmune arthritis development [170]. MiR146a also serves as a negative feedback regulator for B-cell hyperactivation [72]. Collectively, the available evidence shows critical epigenetic regulation during B-cell hyperactivation.

### Epigenetic dysregulation of B cells contributes to autoimmune disease development

Altered B-cell signaling and hyperactivated humoral responses are key drivers of human autoimmunity. Emerging evidence has revealed that epigenetic regulation plays significant roles in B-cell responses, contributing to the initiation and perpetuation of autoimmune diseases. Epigenetic modifications in B cells regulate the selection, activation, and differentiation of autoreactive B cells.

#### Systemic lupus erythematosus

SLE is a systemic autoimmune disease characterized by immune dysregulation, including B-cell hyperactivation and prominent autoantibody production. Recent studies have identified promoter and enhancer site-specific hypomethylation of the CD40L gene in CD4^+^ T cells with disease activity in SLE patients [171, 172]. Evidence has also shown epigenetic dysregulation of B cells in patients with SLE and animal models with SLE-like phenotypes. Further studies have revealed the molecular mechanisms and functional significance of B-cell epigenetic regulation during SLE pathogenesis.

Genome-wide DNA methylation analysis of SLE twin cohorts has shown hypermethylated CpG islands in B cells and identified TNF and EP300 as the most important upstream regulators [173]. Moreover, B cells and other immune populations show marked hypomethylation of interferon-regulated genes, including *IFI44L, PARP9*, and *IFITM1*, which might be associated with disease flares [173]. Scharer et al. studied the transcriptomic and epigenetic programs of circulating B-cell subsets in an African–American cohort with high disease activity. Although the core features of B-cell development are similar, the resting naive B cells from SLE patients are epigenetically distinct from those of healthy controls [12]. Moreover, naive SLE B cells exhibit a unique chromatin architecture. The SLE-specific chromatin accessibility signatures include patterns related to areas surrounding genes that encode transcription factors involved in B-cell activation and differentiation, such as NF-κB, activator protein-1 (AP-1), BATF, IRF4, and Blimp1 [174]. Epigenetic alterations were observed in immature B cells emerging from bone marrow in African–American SLE patients, whereas defects developed at a late stage of B-cell development in European American patients, suggesting that SLE-specific DNA methylation signatures might be ethnicity dependent [175]. Collectively, these data show global epigenetic changes in SLE B cells, which may contribute to B-cell hyperactivation and SLE pathogenesis.

**Fig. 3:**
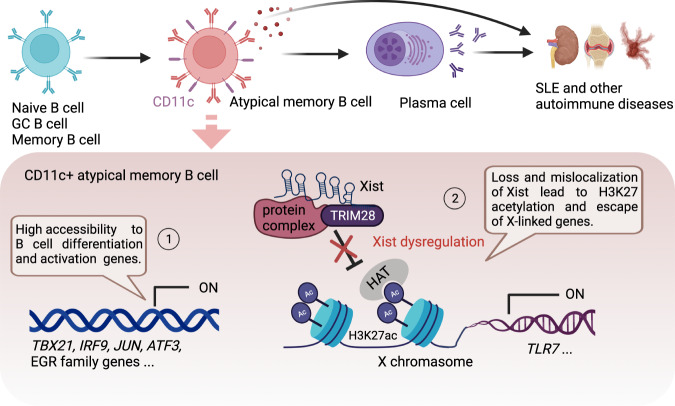
Epigenetic dysregulation of atypical memory B cells contributes to autoimmune diseases. ① Atypical memory B cells from patients with autoimmune diseases, including SLE, show high transcriptional accessibility to genes associated with B-cell differentiation and activation, such as *TBX21, IRF9, JUN, ATF3*, and EGR family genes. ② The lncRNA Xist maintains X-chromosome inactivation (XCI) through continuous deacetylation of H3K27ac at promoters of target genes in the X-chromosome. During autoimmune disease progression, the loss and mislocalization of Xist lead to H3K27 acetylation and escape of X-linked genes such as TLR7 from XCI in atypical memory B cells

Epigenetic changes in functional B-cell subsets are recognized as important factors in SLE development. Although many B-cell subsets show epigenetic alterations, the CpG methylation status identifies the differentiation hierarchies. DN2 (CD27^–^CD11c^+^T-BET^+^CXCR5^–^) B cells and atypical memory B cells show many similarities and are characterized by the abundant expression of CD11c and T-bet. Epigenetic dysregulation of these cells promotes the development of various autoimmune diseases, including SLE (Fig. 3). Atypical memory B cells show more accessible motifs for the T-bet, ISGF3, AP-1, and early growth response (EGR) transcription factor families than other B-cell subsets [12]. Interestingly, atypical memory B cells also show enriched accessible chromatin at T-bet and AP-1 motifs in SWEF-family-deficient mice that develop systemic autoimmunity [176]. SLE DN2 B cells show activation of activating transcription factor 3 (ATF3) response pathways that are induced by BCR and TLR stimulation. The ATF3 and EGR families may act in synergy with T-bet to shape the epigenome of expanded SLE DN2 B cells [12].

The lncRNA Xist sustains XCI during the early development of female cells. It was recently found that B cells from SLE patients showed abnormal XCI with escape of many X-linked genes and Xist RNA interactome genes [13, 107]. Xist is required to suppress the expression of various X-linked immune genes, such as *TLR7*, that play a significant role in human SLE [13, 177]. The escape of TLR7 from XCI increases TLR7 gene products and enhances the B-cell response to TLR7 engagement, which promotes autoantibody production and may drive SLE progression [150]. In particular, scRNA-seq analysis showed Xist dysregulation in CD11c^+^ atypical memory B cells from female SLE patients [13]. Moreover, TLR7 activation and XIST inactivation promote the generation of isotype-switched CD11c^+^ atypical memory B cells [13]. Mechanistically, TRIM28 was identified as a B-cell-specific Xist cofactor [13]. Xist-dependent type I interferon and extrafollicular B-cell pathways are also defective in some SLE patients [178]. Mislocalized Xist RNA predisposes SLE B cells to aberrant X-linked gene expression from the inactivated chromosome and likely contributes to the female bias in SLE [107].

TET enzymes, which are involved in active DNA demethylation, have pleiotropic roles in B-cell homeostasis [68, 69]. A recent study suggests that TET-mediated chromatin modification regulates autoreactive B cells and prevents autoimmunity. Deficiency of *Tet2* and *Tet3* within B cells causes spontaneous B-cell activation and SLE-like disease characteristics, including increased levels of autoantibodies, enhanced proteinuria, and kidney tissue damage [179]. Moreover, Tet2 and Tet3 suppress *Cd86* gene activity dependent on HDAC binding and DNA demethylation at the intron region of *Cd86* [179]. The available evidence suggests that TET proteins participate in the breakdown of B-cell tolerance. Indeed, *TET3* was determined to be a potential SLE susceptibility risk gene by GWAS [6].

Histone modifications participate in regulating B-cell activation and differentiation. Inhibition of HDACs shows therapeutic benefits associated with suppressed B-cell responses in various lupus models. Selective inhibition of HDAC6 by the small molecule ACY-738 decreased the occurrence of glomerulonephritis and the number of plasma cells in New Zealand black × New Zealand white (NZB/W) F1 mice [180]. The HDAC inhibitor panobinostat significantly reduced the number of autoreactive plasma cells and ameliorated nephritis in MRL/lpr mice, whereas other immune populations were largely unaffected [181]. SCFAs exhibit HDAC inhibitor activities. Oral feeding of SCFAs inhibited plasma cell differentiation, suppressed B-cell class switching, and alleviated lupus skin lesions and kidney pathology in both MRL/lpr and NZB/W F1 mice [70]. In contrast, the nonclassical class III HDAC Sirt1 acts as a suppressive epigenetic regulator of *Aicda* and thus regulates SHM during B-cell activation [66]. Sirt1 plays an intrinsic role in modulating autoantibody production during SLE development. The expression of Aicda is negatively correlated with Sirt1 levels in B cells isolated from SLE patients and lupus-prone MRL/lpr mice. *Sirt1* deletion in activated B cells leads to massive production of class-switched autoantibodies, including anti-dsDNA, anti-histone, and anti-ribonucleoprotein antibodies, in normal female C57BL/6 mice [66]. Tightly regulated B-cell activation by HDACs sustains B-cell homeostasis. Breakdown of epigenetic balance may lead to dysregulated B-cell responses and contribute to autoimmunity in SLE pathogenesis.

#### Rheumatoid arthritis

RA is a common chronic autoimmune disease characterized by immune dysregulation and progressive joint damage. B cells are one of the most important players in RA development. Massive infiltration of B cells and plasma cells is observed in the synovial tissues of RA patients. Importantly, more than 80% of RA patients are seropositive for autoantibodies, including rheumatoid factor and anti-citrullinated protein antibodies (ACPAs), which further highlights the significant roles of B cells in RA [182].

Epigenetic changes in RA patients have been studied in various immune and nonimmune populations. Aberrant DNA methylation and histone modification patterns and miRNA expression levels are considered important contributors to RA pathogenesis [183]. An epigenome-wide association study (EWAS) of RA patients with three different replication cohorts identified several important disease-specific alterations of DNA methylation in B cells [184]. DNA hypermethylation at *CD1C* and hypomethylation at *TNFSF10* genes are associated with elevated RA risk. Casitas B-lineage lymphoma (CBL) proteins interact with BCR downstream signaling factors and control the B-cell-intrinsic checkpoint of immune tolerance [185]. B cells from RA patients show differential methylation at the genes involved in CBL pathways, which may have functional significance in RA pathogenesis. These epigenetic alterations are also detected in SLE patients, suggesting that patients with these two conditions have shared methylation alterations [184]. There is a potential impact of therapeutic treatments on epigenetic factors, but genome-wide profiling revealed a novel methylation signature related to 113 sites within B cells in treatment-naive early RA patients [186]. Both early and established RA patients show similar DNA methylation patterns in B cells [186].

Small RNA sequencing of B cells revealed 27 miRNAs that are differentially expressed in MTX-treated RA patients compared with healthy controls. However, no significant differences were observed between newly diagnosed RA patients and healthy donors [187]. The differentially expressed miRNA target genes involved in B-cell activation, differentiation, and BCR signaling included *STAT3, PRDM1*, and *PTEN*. miRNA-155 is a critical regulator of GC and plasma cell responses [85]. Mice with miR-155 deficiency are resistant to CIA, with significantly suppressed antigen-specific Th17-cell and autoantibody responses and markedly reduced joint inflammation [188]. Moreover, the expression levels of miRNA-155 are increased in B cells from RA patients compared with the levels in B cells from healthy individuals. In particular, miRNA-155 is highly expressed in IgD^-^CD27^-^ memory B cells from ACPA-positive RA patients. In synovial tissues, miRNA-155 expression is negatively associated with PU.1 expression in B cells. Inhibition of endogenous miRNA-155 in B cells restores PU.1 expression and decreases antibody production, suggesting that miRNA-155 modulates B-cell functions by suppressing PU.1 in RA patients [189].

#### Primary Sjögren’s syndrome

As a chronic autoimmune disorder, pSS is characterized by inflammation and tissue destruction in salivary glands and lacrimal glands, which cause dry mouth and dry eyes. Although many immune cell populations and cytokines are involved in pSS development, clinical observations support a critical role of B cells in pSS pathogenesis since patients have diverse autoantibodies and increased risks of developing B-cell lymphoma [7, 190–192]. Autoantibodies specific for pSS may even develop long before symptoms emerge, suggesting a key role of B cells in disease initiation and perpetuation. GWASs have revealed risk genes associated with B-cell activation, such as *BLK*, CXCR5, and *PRDM1* [7]. An epigenetic analysis showed that DNA methylation changes are mainly present in B cells but not in T cells. Moreover, genes with differentially methylated probes overlapped with the risk genes identified by GWAS, including *BLK* and CXCR5, suggesting genetic and epigenetic dysregulation of these key genes during pSS development [193]. Interferon-regulated genes are hypomethylated in B cells and are associated with increased B-cell numbers [193, 194]. Methylation changes in these genes in B cells are positively correlated with disease activity, further emphasizing the central role of B cells and the relevance of DNA methylation changes in pSS pathogenesis [193, 194].

A genome-wide case‒control study revealed many differentially methylated areas in inflamed minor salivary gland biopsies from pSS patients. Although this study used a mixture of epithelial cells and infiltrating immune cells, the enrichment analysis showed dysregulated epigenetic control of genes associated with B-cell survival and functions, such as *CXCR5* and *TNFRSF13B* [195].

B cells from pSS patients and healthy controls show major differences in miRNA expression patterns. Several differentially expressed miRNAs, including hsa-mir-30b-5p, have been identified in B cells. Inhibiting hsa-mir-30b-5p increases BAFF expression, suggesting a functional role of the miRNA in B-cell responses [196]. A transcriptomic analysis showed increased expression of the lncRNA LINC00487 in various B-cell subsets from pSS patients, which was correlated with disease activity scores [197]. These studies show dysregulated epigenetic control of B cells in pSS patients, highlighting an important role of B cells during pSS development.

#### Multiple sclerosis

MS is an autoinflammatory and neurogenerative disease that leads to demyelination of neurons and axonal loss in the central nervous system (CNS). Although MS has been traditionally considered a T-cell-dominated autoimmune disease, recent studies in the past decade have revealed the critical involvement of B cells during MS pathogenesis [198]. Abnormal cytokine profiles in B cells have been observed in MS patients. These B cells produce an excessive amount of proinflammatory cytokines, including TNF, IL-6, and GM-CSF [198]. B cells may directly target oligodendrocytes and neurons by producing cytotoxic mediators. Furthermore, the interplay between B and T cells may have a pivotal role since it has been shown that B cells drive the proliferation and migration of brain-homing, autoreactive CD4 T cells in MS [199]. Clinical trials show the therapeutic benefits of B-cell depletion therapies in many MS patients, supporting the role of B cells in MS pathogenesis [200].

Compared with monocytes and T cells, B cells display the most significant differences in methylated areas, showing a global DNA hypomethylation signature [201, 202]. In a cohort involving 24 patients with relapsing-remitting MS and 24 healthy controls, EWAS analysis of DNA methylation also revealed extensively altered methylation patterns in B cells of MS patients [203]. The genes with differentially methylated areas were found to be involved in innate immunity and BCR signaling. Notably, hypermethylated areas were identified around the transcriptional start site of the *LTA* gene, which encodes the proinflammatory cytokine lymphotoxin alpha secreted by B cells and other immune cells [203]. These epigenetic features highlight specific epigenetic programs involved in B-cell differentiation, activation, and function.

#### Systemic sclerosis

Systemic sclerosis (SSc) is a severe autoimmune disease characterized by vascular damage and immune dysregulation that leads to fibrosis of the skin and internal organs. Although genetic susceptibility is important in SSc, epigenetic modifications constitute the driving force for disease initiation [204]. Environmental factors such as exposure to silica or viruses can trigger epigenetic modifications and are linked to genetic susceptibility [204]. Genetic and epigenetic factors ultimately cause a series of cellular processes that lead to tissue fibrosis. Altered DNA methylation and histone modification profiles and dysregulated levels of noncoding RNAs together contribute to the development of SSc. Although it has been clear that genetic predisposition can only partially explain SSc occurrence, cell-specific epigenetic regulation during SSc is not fully understood.

B cells, T cells, monocytes, and fibroblasts show altered histone modification signatures in SSc patients compared with healthy controls, suggesting a role of epigenetic events in immune dysregulation and fibrogenesis. Activated B cells can affect tissue fibrosis through diverse effector mechanisms in SSc [205]. Notably, B-cell depletion inhibits tissue fibrosis by suppressing profibrotic macrophage differentiation in a mouse model of SSc, further supporting an important role of B cells in the pathogenesis of SSc [206]. In SSc patients, global histone H4 hyperacetylation and histone H3K9 hypomethylation are detected in B cells. Moreover, H4 acetylation levels are negatively correlated with HDAC2 expression but positively correlated with disease activity, suggesting a direct association between B-cell epigenetic alterations and clinical disease severity in SSc patients [205]. In addition to B cells, monocytes have altered histone modifications correlated with an enhanced IFN signature in SSc patients [207]. Interestingly, a genomic methylation study of whole blood from SSc discordant twin pairs showed shared methylated sites between SSc and SLE patients across various immune populations, suggesting that these epigenetic features may be common biomarkers for autoimmune diseases [208].

#### Type 1 diabetes

T1D is one of the most common autoimmune diseases and is characterized by the destruction of insulin-producing β cells in the pancreas. Although previous studies showed a dominant role of T cells in pancreatic tissue destruction, accumulated evidence suggests that T1D is associated with the tolerance breakdown of B cells [209]. These autoreactive B cells contribute to T1D development by producing autoantibodies and presenting autoantigens to T cells. Importantly, recent studies suggest that the generation of islet-reactive B cells is associated with certain genetic polymorphisms and aberrant epigenetic events [209, 210].

Epigenetic studies of monozygotic twins discordant for T1D have been performed to investigate the effects of nongenetic factors during disease development. A genome-wide DNA methylation study identified 88 CpG sites displaying significant methylation changes in B cells from 3 pairs of T1D-discordant monozygotic twins [211]. Functional annotation suggested that these epigenetic changes may be associated with antigen presentation by B cells [211]. In a larger EWAS, differentially variable CpG positions were detected across 406,365 CpGs in CD4 T cells, B cells, and monocytes in 52 monozygotic twin pairs discordant for T1D [212]. Twins with T1D showed substantial enrichment of differentially variable CpG positions compared with their healthy twin and unrelated healthy individuals [212]. Evidence from studies of cord blood of newborns who developed T1D suggests that these CpG modifications are likely to emerge after birth [212]. An analysis of cell type-specific gene regulatory circuits highlighted immunometabolism and mTOR signaling as enriched pathways in B cells [212]. T1D-associated genetic and epigenetic variants seem to act independently since the differentially variable CpG positions are not enriched in genetic susceptibility loci [212].

## Epigenetic biomarkers and therapies for autoimmune diseases

### Epigenetic biomarkers for autoimmune diseases

Current diagnostic criteria for autoimmune diseases have been developed largely based on clinical manifestations and laboratory tests, and there is a lack of biomarkers with high sensitivity and specificity [213]. The shared clinical parameters among different autoimmune diseases and heterogeneity of the patients highlight an essential need for novel biomarkers that can reflect disease characteristics and predict therapy responses. Epigenetic changes are considered one of the earliest factors associated with disease initiation before clinical manifestations. There has been rapid development of molecular biology techniques, and increasing evidence derived from such techniques shows that abnormal epigenetic events may serve as promising biomarkers for autoimmune diseases.

Genome-wide DNA methylation studies have already revealed differentially methylated sites in patients with autoimmune diseases, including SLE, RA, and SSc [14]. Integrative analysis of a multiple autoimmune disease methylation dataset revealed that hypomethylation of IFN-related genes is a common feature of RA, SLE, and SSc, suggesting that the DNA methylation profile of IFN-related genes could be a biomarker for the diagnosis of autoimmune diseases [14]. In SLE patients, altered epigenetic modifications support the dysfunction of various B-cell subsets, such as DN2 B cells [12]. Importantly, DNA methylation profiling studies have identified distinct molecular signatures of differentially methylated loci that could stratify healthy controls and SLE patients [12]. These signature loci include hypomethylated sites near interferon-induced genes such as *IFI44* and *IFITM1* and hypermethylated CpGs surrounding *SOX12, ARFGAP3, CCDC81*, and *MEG3*. The DNA methylation patterns in B cells from healthy controls and SLE patients show clear differences, suggesting that the identified differentially methylated loci could be used as SLE biomarkers [12]. Since altered DNA methylation modifications are detected in diverse B-cell subsets, the associations between epigenetic modifications and disease status deserve further investigation. Synovial and blood monocytes of undifferentiated arthritis patients show marked alterations in methylation profiles compared with those from healthy donors [15]. DNA methylation-based biomarkers are closely associated with prognosis, disease activity, and treatment efficacy in patients with early inflammatory arthritis, suggesting that the DNA methylation signatures of many immune subsets could serve as important biomarkers for personalized clinical management of autoimmune disease patients [15, 16].

It has been well characterized that type I IFN pathways are activated and involved in many autoimmune diseases, including SLE. Increased type I IFN levels are observed in and associated with nephritis, mucocutaneous manifestations, and the presence of autoantibodies in SLE patients [214]. The production of type I IFNs is triggered by the activation of nucleic acid-binding pattern recognition receptors, including TLR7, cyclic GMP-AMP synthase, and RIG-I-like receptors. Several genetic and epigenetic studies have shown a clinical association of aberrant IFN-stimulated gene expression with SLE development [214]. Notably, SLE patients show significant hypomethylation of two CpG sites within the promoter region of *IFI44L*, a typical type I interferon-stimulated gene, when compared with healthy controls, RA patients, and pSS patients [215]. In a paired analysis of twins discordant for SLE, B cells, T cells, and monocytes in the diseased twin all exhibited significant hypomethylation of interferon-stimulated genes, including *IFI44L*, which was even more pronounced in twins who experienced a disease flare within the past 2 years [173]. In addition, the methylation levels around the *IFI44L* promoter are negatively associated with renal damage. The DNA methylation status at the two *IFI44L* promoter sites has higher sensitivity and specificity than most available tests, suggesting that the methylation level of *IFI44L* is a highly sensitive and specific diagnostic marker for SLE [215]. Hypomethylation at other IFN-stimulated genes, such as *IFI44* and *IFITM1*, in B cells also discriminates SLE patients from healthy controls, providing further evidence that DNA methylation around IFN-stimulated genes is a promising biomarker for SLE [12]. Interestingly, bioinformatic analysis and machine learning have identified IFI44 as an optimal diagnostic marker of SLE, which was verified by quantitative real-time PCR in an independent cohort [216]. Genome-wide DNA methylation profiling also revealed prominent hypomethylation of interferon-stimulated genes such as *MX1*, *IFI44L*, and *PARP9* in B cells from pSS patients, suggesting that methylation levels at different IFN-stimulated gene loci may be biomarkers for different autoimmune diseases [194].

Regarding histone modifications, early studies showed significant alterations of H3K4me3 in peripheral blood mononuclear cells (PBMCs) of SLE patients and H3K9 hypomethylation in SSc patients [205, 207, 217]. These histone methylation changes are associated with disease activity and may be clinical and biological markers.

Increasing evidence suggests the strong involvement of noncoding RNAs in B-cell responses and autoimmunity. Many studies have shown a particularly important role of aberrant noncoding RNAs, including miRNAs, in autoimmune disease pathogenesis, which suggests that noncoding RNAs could serve as promising biomarkers for various autoimmune diseases [218]. The expression levels of miRNAs are relatively stable in serum and thus could be assessed in a reproducible and consistent manner. miRNAs can be detected in various tissues in a rapid and precise way, showing promising clinical application potential.

Genome-wide miRNA expression profiling has revealed significantly altered miRNA signatures in naive and memory B cells from renal and nonrenal severe SLE patients of Latin American background. B-cell subset-specific miRNAs were characterized in healthy donors and SLE patients. Specific miRNA signature profiles could be applied to discriminate naive and memory B cells of SLE patients from those of healthy controls. Moreover, six miRNAs were found to be associated with specific pathologic features representing renal outcomes in SLE patients, suggesting that they are promising biomarkers for molecular diagnosis. Many miRNAs have been shown to have an abnormal expression in serum, PBMCs, T cells, and tissue cells from patients with SLE as well as other autoimmune diseases [213], suggesting that combined miRNA signatures in various organs and cell populations may provide precise clues for the diagnosis and clinical management of autoimmune diseases. SLE B cells show aberrant expression and localization of the lncRNA Xist. In particular, Xist-mediated escape of many X-linked genes, including TLR7, contributes to dysregulated B-cell responses and SLE development [13, 107]. SLE T cells also show altered expression of *XIST* RNA interactome genes, showing dysregulated Xist-associated XCI expression in diverse immune cells [219, 220]. Xist is aberrantly expressed, and Xist expression is associated with disease activity, clinical manifestations, and laboratory parameters of SLE, suggesting that Xist might be a biomarker for SLE [219–221].

### Targets for epigenetic therapy

#### EZH2

EZH2 is a histone methyltransferase subunit of the polycomb repressive complex that mediates trimethylation of Lys27 in histone H3. EZH2 plays an important role in regulating B-cell differentiation and metabolism during the GC reaction and plasma cell formation [93, 94]. EZH2 also controls T-cell functions in cooperation with transcription factors and has been proposed as a novel therapeutic target for treating SLE patients [222].

In SLE patients, the expression of EZH2 is elevated and positively correlated with the overexpression of IFN-stimulated genes in PBMCs and renal tissues of SLE patients [223].

Notably, increased EZH2 expression is detected in whole blood, neutrophils, monocytes, B cells, and CD4+ T cells [224]. Treatment with the EZH2 inhibitor GSK126 prolonged survival time, reduced the levels of anti-dsDNA autoantibodies, and improved lupus nephritis in NZB/W F1 mice [223]. Moreover, an EZH2 inhibitor decreased the expression of IFN-stimulated genes in the kidneys of these mice, suggesting that EZH2 inhibition interferes with the activation of type I IFN signaling pathways [223]. In lupus-prone MRL/lpr mice, intraperitoneal administration of 3-deazaneplanocin A (DZNep), an EZH2 inhibitor, improved survival and significantly reduced anti-dsDNA antibody levels. DZNep-treated mice exhibited a significant reduction in renal damage, splenomegaly, and lymphadenopathy with a decrease in the levels of many cytokines and chemokines [224]. In an allogeneic T-cell-induced lupus model, deficiency or pharmacological inhibition of EZH2 suppresses GC formation and autoantibody production [225]. Increased expression of EZH2 seems to be associated with metabolic disorders since mTORC1-mediated glycolysis controls EZH2 expression in SLE T cells, suggesting that glycolytic pathways might be indirect targets for suppressing EZH2 expression [226]. In addition to SLE, EZH2 is involved in other autoimmune diseases, including autoimmune hepatitis. High expression of EZH2 promotes immune activation and liver fibrosis through H3K27me3 whereas DZNep treatment attenuates hepatic inflammation and liver fibrosis in mice with autoimmune hepatitis [227]. Currently, supportive evidence from clinical trials on the efficacy of EZH2 inhibitors in autoimmune patients is still lacking. However, active clinical investigations of EZH2 inhibitors in patients with cancer will shed new light on the future development of EZH2-targeted therapies for patients with autoimmune diseases.

#### HDACs

Histone acetylation balance has shown major effects on chromatin remodeling and gene transcription. Histone acetylation and deacetylation are catalyzed by HATs and HDACs, respectively. HDACs are important for B-cell development and activation. In recent decades, many HDAC inhibitors have been developed and have shown therapeutic potential for treating autoimmune diseases.

In MRL/lpr mice with SLE-like phenotypes, HDAC6 is highly expressed in B cells. A selective HDAC6 inhibitor, ACY-738, inhibits pre-B-cell proliferation by regulating Bax-mediated pathways [228]. Treatment with ACY-738 also significantly decreased proteinuria scores and prevented lupus nephritis in NZB/W F1 mice. Moreover, ACY-738 administration dramatically reduces the numbers of GC B cells and plasma cells, leading to a reduction in serum autoantibody levels and attenuated IgG deposition in the glomerulus [229]. A novel HDAC6-selective inhibitor, CKD-506, exerts prophylactic and therapeutic effects in experimental autoimmune encephalomyelitis (EAE) mice by regulating peripheral immune responses and maintaining blood‒brain barrier integrity [230]. Another HDAC inhibitor, panobinostat, which targets class I, II, and IV HDACs, ameliorates renal damage and decreases disease severity while significantly reducing plasma cell responses and autoantibody levels in MRL/lpr mice [181]. However, other immune parameters are largely unaffected [181]. Interestingly, panobinostat has shown promising therapeutic efficacy for treating multiple myeloma, a B-cell malignancy [231].

Dysregulated expression of HDACs is detected in synovial tissues from RA patients and linked to disease pathology [232]. Although the functions of HDACs in immune tolerance breakdown and joint damage are not fully understood, preclinical studies have shown that HDAC inhibitors exert both prophylactic and therapeutic benefits [232]. For example, MPT0G009 shows potent inhibitory effects on various HDAC isoforms and inhibits the development of arthritis in an adjuvant-induced arthritis model [233]. A well-studied HDAC inhibitor, givinostat, exhibits significant therapeutic benefit and good safety profiles in patients with systemic-onset juvenile idiopathic arthritis [234]. The available studies suggest that HDAC inhibitors exhibit major effects on synoviocytes and T cells in arthritic mice [232]. Further studies are needed to investigate the effects of HDAC inhibitors on B-cell-mediated arthritis development in mice and patients with RA.

Many HDAC inhibitors have been developed, such as panobinostat, entinostat, and ricolinostat. Some of these agents have been tested in clinical trials for patients with cancers. Apart from small molecules that target HDACs, recent studies suggest that natural metabolites may also serve as HDAC inhibitors. SCFAs directly modulate plasma cell differentiation and antibody production. By acting as HDAC inhibitors, SCFAs significantly decrease AID and Blimp1 expression in B cells, reduce serum levels of class-switched autoantibodies, and abolish lupus-like skin lesions and kidney pathology in both MRL/lpr and NZB/W F1 mice, suggesting that dietary fibers and related catabolites produced by intestinal microbiota may exert beneficial effects for ameliorating autoimmunity [70].

Hundreds of clinical trials associated with HDAC inhibitors have been registered, most of which are designed to evaluate safety and efficacy in patients with different cancers, inflammatory diseases, and neurodegenerative disorders. Although available clinical evidence of HDAC inhibitors in autoimmune disease patients is limited, studies on immune system disorders will provide more clues and evidence for future investigations.

#### DNMTs

DNMTs are a conserved family of cytosine methyltransferases. DNMTs and TETs sustain a homeostatic balance of DNA methylation. It has been reported that both DNMT3A and DNMT3B suppress the expansion of GC B cells and plasma cells [81] and may control B-cell lineage-specific gene expression [125]. As discussed, DNA methylation signatures may serve as promising biomarkers for the diagnosis and clinical management of SLE and other autoimmune diseases. Some currently used immunosuppressive drugs for patients with autoimmune diseases can induce epigenetic changes, which could partially account for their therapeutic effectiveness [11]. In mice with EAE, low-dose treatment with 5-aza-2’-deoxycytidine (5-azadC), an inhibitor of DNMTs, increases the immunosuppressive function of regulatory T cells, suppresses CNS inflammation, and inhibits EAE development [235]. Similarly, low-dose treatment with 5-azacytidine (5-azaC), another DNMT inhibitor, reduces disease severity and halts joint inflammation in mice with proteoglycan-induced arthritis [236]. Moreover, 5-azaC treatment compromises GC formation, leading to suppressed SHM, CSR, and IgG1 antibody production [236]. These findings suggest that inhibition of DNMTs by pharmacological small molecules can modulate B-cell and T-cell responses and provide therapeutic benefits in mouse models of autoimmune diseases. 5′-Azacytidine has been approved for the treatment of several blood cell malignancies, suggesting the therapeutic potential of DNMT inhibitors in autoimmune diseases. However, it is important to note that DNMT inhibition by 5-azadC may also increase antibody production by B cells, indicating complex effects on disease development under different conditions [11]. Targeting DNMTs in specific cell types is important since different cell subsets show distinct DNA methylation features. Inhibiting DNMTs in CD4 and CD8 T cells with 5-azaC and a nanolipogel delivery system dramatically ameliorates lupus-related pathology through distinct mechanisms. Targeted inhibition of DNMTs in CD4 T cells promotes Treg expansion, whereas inhibiting DNMTs in CD8 T cells restrains pathogenic CD4^-^CD8^-^ double-negative T cells in MRL/lpr mice, indicating the significance of cell-specific inhibition of DNA methylation [237]. Since hypomethylation of IFN-related genes is common in B cells and other immune cells from patients with SLE or SSc [14], inhibiting DNA methylation may not be an optimal therapeutic approach. In diseases with global hypomethylation, future investigations to explore novel agents that can specifically increase DNA methylation in B cells and other populations without triggering severe side effects are important.

#### miRNAs

Various miRNAs have been identified as important regulators during B-cell activation and the development of various autoimmune diseases [38]. Dysregulated miRNA profiles are involved in the pathogenesis of autoimmune diseases via diverse effector mechanisms, including regulating immune cell activation, cytokine release, and autoantibody production [218]. Moreover, miRNA signature profiles may serve as useful clinical biomarkers. Preclinical studies have shown that miRNAs may also be therapeutic targets. B cells from MRL/lpr mice show high levels of miR-7, which directly targets *Pten* mRNA. Treatment with a miR-7 antagomir significantly reduces hypertrophy of the spleen, decreases proteinuria, and inhibits immune complex deposition in glomeruli in MRL/lpr mice. Notably, a miR-7 antagonist normalized B-cell hyperresponsiveness, suggesting that inhibition of miR-7 alleviates lupus nephritis by modulating B-cell responses [238]. MiR-155 is important in GC and plasma cell responses and is associated with RA development [85, 188, 189]. Moreover, administration of a miR-155 antagomir attenuates pristane-induced lupus alveolar hemorrhage in mice [239]. Treatment with an antisense oligonucleotide that targets miR155 reduces CNS inflammation and decreases the disease severity of EAE mice before and after the appearance of clinical symptoms [240], suggesting that miR-155 may represent an attractive therapeutic target.

Recently, many miRNA-based therapies have been developed. Compared with other biological agents, such as monoclonal antibodies, miRNAs show unique advantages in terms of drug development. miRNAs are often conserved among species with known sequences. Therefore, it is possible to deliver synthetic oligonucleotides or small molecules that can either supplement or block the effects of target miRNAs. The available platforms for delivering therapeutic oligonucleotides provide a solid foundation for miRNA-based therapies. However, since miRNAs can regulate various targets during different cellular processes, the incomplete understanding of miRNA biology may restrain translational studies of miRNA therapies in the clinic due to potential off-target and side effects [241]. Currently, the clinical application of miRNA-based therapies is at an early stage. Although available evidence shows promising therapeutic potential of epigenetic therapies (Table 1), further clinical trials will be needed for the validation of novel therapeutic strategies in patients with autoimmune diseases.

**Table 1:** Epigenetic targets for autoimmune diseases

Targets	Agents	Diseases/models	Therapeutic results
EZH2	GSK126	NZB/W mice	Increased survival time, reduced levels of anti-dsDNA autoantibodies, and improved lupus nephritis [223]
	DZNep	MRL/lpr mice	Improved survival rates and reduced anti-dsDNA antibody levels [224]
	GSK503	Murine bm12 model of lupus-like chronic graft versus host disease	Suppressed germinal center formation and autoantibody production [225]
	DZNep	Induced autoimmune hepatitis mice	Attenuated hepatic inflammation and liver fibrosis [227]
HADCs	ACY-738	NZB/W mice	Decreased proteinuria scores and serum autoantibodies levels, attenuated IgG deposition in glomerulus, and ameliorated lupus nephritis [229]
	CKD-506	EAE mice	Decreased clinical scores and central nervous system inflammation [230]
	Panobinostat	MRL/lpr mice	Ameliorated renal damage, reduced autoantibody levels, and decreased disease severities [181]
	MPT0G009	Adjuvant-induced arthritis mice	Ameliorated joint inflammation and cartilage damage [233]
	Givinostat	Systemic-onset juvenile idiopathic arthritis patients	Significant therapeutic benefit and tolerated safety profiles in the patients [234]
	SCFAs	MRL/lpr mice and NZB/W mice	Reduced serum levels of class-switched autoantibodies, abolished lupus-like skin lesions and kidney pathology [70].
DNMTs	5-azadC	EAE mice	Suppressed central nervous system inflammation [235]
	5-azaC	Proteoglycan-induced arthritis mice	Reduced disease severity and halted joint inflammation [236]
	5-azaC	MRL/lpr mice	Ameliorated lupus-related pathology [237]
miRNAs	miR-7 antagomir	MRL/lpr mice	Reduced spleen hypertrophy, decreased proteinuria, and inhibited immune complex deposition in glomeruli [238]
	miR-155 antagomir	CIA mice	Reduced disease severity [245]
	miR-155 antagomir	Pristane-induced lupus mice	Attenuated pristane-induced lupus alveolar hemorrhage [239]
	miR-155 antagomir	EAE mice	Reduced central nervous system inflammation and decreased disease severities [240]

*DZNep* 3-Deazaneplanocin A, *EAE* experimental autoimmune encephalomyelitis, *CIA* collagen-induced arthritis, *SCFAs* short-chain fatty acids

## Challenges and future perspectives

### The epigenome in B-cell subsets and its roles in autoimmunity

Mounting evidence has shown the important roles of epigenetic alterations in many B-cell subsets and B-cell-mediated autoimmunity. During B-cell activation and differentiation, there is a dramatic change in the epigenome that involves diverse effector mechanisms, including those related to DNA methylation, histone modifications, and noncoding RNAs. With the rapid development of genetic approaches, the roles of epigenetic processes can be studied using mice with B-cell-conditional deficiency of key enzymes. However, the roles of epigenetic events in B-cell subsets can hardly be determined using these genetic methods. Since B cells may exert different functions under different disease conditions due to cellular heterogeneity, it is important to investigate the epigenomes of functional B-cell subsets.

Plasma cells are terminally differentiated B cells that exert diverse functions by producing antibodies as well as cytokines such as IL-10 [5]. It has been well recognized that plasma cells play crucial roles in the development of many autoimmune diseases. The differentiation of plasma cells is tightly regulated by intrinsic transcription factors and the surrounding microenvironment [242]. Although recent studies suggest that HDACs, DNMTs, and TETs contribute to plasma cell generation, it remains largely unclear how epigenetic events sustain plasma cell identity and regulate plasma cell functions. Atypical memory B cells serve as a key pathogenic player in autoimmune diseases. Dysregulated epigenetic events, including DNA hypomethylation and mislocated Xist, have been identified in SLE patients [12, 13]. Although the available evidence suggests a role for epigenetic alterations in the formation of atypical memory B cells, further investigations on the detailed mechanisms and functions of other epigenetic events in these cells are warranted. Future studies on epigenetic regulation in functional B-cell subsets will further enhance the understanding of autoimmune pathogenesis and may provide novel therapeutic targets for treating patients.

### Epigenetics for clinical therapy and personalized medicine

Over the past years, there has been enormous progress in genome-wide epigenetic studies with fruitful achievements. The epigenetic signatures in patients with autoimmune diseases may be novel biomarkers for clinical diagnosis and may provide new epigenetic targets for therapies [10]. Targeting epigenetic modifications may have side effects since epigenetic events usually have tissue-specific and cell-specific functions. Although many clinical trials have been performed on cancer patients, limited clinical evidence has been obtained regarding the safety and effectiveness of epigenetic therapies in patients with autoimmune diseases. The insufficient understanding of epigenetic mechanisms in disease pathology and the side effects of current epigenetic therapies are major challenges for clinical applications.

The personalized treatment of cancer patients by integrating genomic information and other data, including clinical profiles and epigenetic results, has been proposed [243]. Personalized medicine has been extensively discussed in the context of cancer treatment and is receiving increasing attention in the field of autoimmune diseases. Patients usually show variable responses to the same therapy, suggesting heterogeneity of patients and the need for more precise therapies. Available evidence suggests that epigenomic profiles are not only important for understanding autoimmune disease pathogenesis but also valuable for evaluating disease progression and may provide clues for predicting responses to specific therapies [16, 213, 244].

Many studies suggest that epigenetic signatures may serve as useful biomarkers for the diagnosis and clinical management of patients [213]. As previously discussed, several studies have revealed significant epigenetic changes around genes associated with type I IFN and identified potential diagnostic markers for SLE [14, 213, 215, 216]. In addition to the global DNA methylation profiles in B cells and other cell populations, methylation levels of circulating DNA and concentrations of circulating miRNAs in peripheral blood have been detected in SLE patients and might be a potential indicator of disease outcome [10].

The availability of low-cost methods for detecting epigenomic changes and emerging bioinformatic tools have allowed the rapid development of strategies for genome-wide epigenetic analysis of different immune cell subsets. Together with genomic studies such as GWAS and scRNA-seq, global epigenetic studies will reveal the detailed gene expression landscape in various populations, which will provide further insights into disease pathogenesis and facilitate the discovery of new biomarkers and the development of novel personalized therapies.

## Conclusions

Current evidence demonstrates the important roles of both genetic and epigenetic factors in the immunopathogenesis of autoimmune diseases. Genome-wide DNA methylation analysis and B-cell lineage-specific genetic approaches have revealed the significant role of epigenetic modifications in various functional B-cell subsets and the participation of epigenetic alterations in the development of autoimmune diseases. However, the underlying mechanisms by which epigenetic alterations affect different B-cell subsets during diverse disease conditions need further investigation. Comparative studies between patients and healthy populations have identified important B-cell epigenetic signatures, which could be novel biomarkers of disease prognosis and severity and responses to therapies. Unlike genetic changes, epigenetic alterations are inherently reversible, making them attractive therapeutic targets. Preclinical studies show that inhibitors of EZH2, HDACs, DNMTs, and some miRNAs exhibit therapeutic benefits in animal models. Currently, clinical trials of epigenetic therapies in patients with autoimmune diseases are still lacking. The characterization of the B-cell epigenetic landscape by high-throughput technologies will provide further insights into disease pathogenesis, facilitate the discovery of novel biomarkers, and promote the development of personalized therapies for patients with autoimmune disorders.
